# Parental Involvement Policies for Minors Seeking Abortion in the Southeast and Quality of Care

**DOI:** 10.1007/s13178-021-00539-0

**Published:** 2021-02-13

**Authors:** Kari White, Subasri Narasimhan, Sophie A. Hartwig, Erin Carroll, Alexandra McBrayer, Samantha Hubbard, Rachel Rebouché, Melissa Kottke, Kelli Stidham Hall

**Affiliations:** 1 Steve Hicks School of Social Work and Department of Sociology, University of Texas at Austin, TX, Austin, USA; 2 Department of Behavioral, Social, and Health Education Sciences and Center for Reproductive Health Research in the Southeast, Emory University, Atlanta, GA, USA; 3 Department of Health Care Organization and Policy, University of Alabama at Birmingham, AL, Birmingham, USA; 4 ICF, Inc, Atlanta, GA, USA; 5 Beasley School of Law, Temple University, Philadelphia, PA, USA; 6 Department of Gynecology and Obstetrics and Jane Fonda Center, Emory University, Atlanta, GA, USA; 7 Heilbrunn Department of Population & Family Health, Columbia University Mailman School of Public Health, New York City, NY, USA

**Keywords:** Abortion, Healthcare quality, Minors, Parental involvement laws, United states

## Abstract

**Introduction:**

Thirty-seven states require minors seeking abortion to involve a parent, either through notification or consent. Little research has examined how implementation of these laws affect service delivery and quality of care for those who involve a parent.

**Methods:**

Between May 2018 and September 2019, in-depth interviews were conducted with 34 staff members involved in scheduling, counseling, and administration at abortion facilities in three Southeastern states. Interviews explored procedures for documenting parental involvement, minors’ and parents’ reactions to requirements, and challenges with implementation and compliance. Both inductive and deductive codes, informed by the *Institute of Medicine’s* healthcare quality framework, were used in the thematic analysis.

**Results:**

Parental involvement laws adversely affected four quality care domains: efficiency, patient-centeredness, timeliness, and equity. Administrative inefficiencies stemmed from the extensive documentation needed to prove an adult’s relationship to a minor, increasing the time and effort needed to comply with state reporting requirements. If parents were not supportive of their minor’s decision, participants felt they had a duty to intervene to ensure the minor’s decision and needs remained centered. Staff further noted that delays to timely care accumulated as minors navigated parental involvement and other state mandates, pushing some beyond gestational age limits. Lower income families and those with complex familial arrangements had greater difficulty meeting state requirements.

**Conclusions:**

Parental involvement mandates undermine health service delivery and quality for minors seeking abortion services in the Southeast.

**Policy Implications:**

Removing parental involvement requirements would protect minors’ reproductive autonomy and support the provision of equitable, patient-centered healthcare.

## Introduction

Parental involvement laws for minors are among the most long-standing state-level restrictions on abortion care. Currently, 37 states require minors seeking abortion to involve a parent, with 21 mandating parental consent, 11 requiring parental notification, and 5 requiring both consent and notification; both parents are required to provide consent in three states, and in one state, both parents must be notified (Guttmacher Institute, 2020a). Although the US Supreme Court has ruled that states must allow minors an alternative to parental involvement, that process, which typically takes the form of a court hearing known as a judicial bypass, can be intimidating and challenging for minors and may discourage them from pursuing that option (K. T. Coleman-Minahan et al., 2019; Kavanagh et al., 2012). As such, bypasses are used by only a small percentage of minors obtaining abortion (Altindag & Joyce, 2017; MacAfee et al., 2015).

Prior studies of parental consent and notification laws have largely examined their impact on the gestational age at which minors obtain an abortion and the extent to which minors seek abortion care in less restrictive states (Dennis et al., 2009; Janiak et al., 2019; MacAfee et al., 2015). Even though most minors involve a parent in their care (Hasselbacher et al., 2014; Ralph et al., 2018), there has been limited research on how parental involvement laws are implemented by abortion facilities and the ways in which the laws affect service delivery and quality of care for minors who have to comply with these mandates. In a review of abortion care safety and quality, the National Academies of Science, Engineering, and Medicine concluded that abortion restrictions often adversely affect the Institute of Medicine’s (IOM) six domains of quality of care: safety (avoiding harm), effectiveness (providing care grounded in science), efficiency (reducing waste of materials and human energy), patient-centeredness (incorporating patient preferences), timeliness (avoiding delays), and equity (ensuring quality does not differ by patient characteristics) (National Academies of Sciences, Engineering, and Medicine, 2018). These effects on quality care likely also extend to parental involvement policies and the quality of care for minor patients. As parental involvement laws have become more restrictive in some states (e.g., requiring parents to provide a notarized statement or proof of parenthood), and other states move to ease their requirements (Nadie, 2020), an in-depth assessment of how these laws affect quality care is needed.

In consideration of these domains of healthcare quality (Institute of Medicine & Committee on Quality of Health Care in America, 2001), we explore staff perspectives and experiences complying with parental involvement laws at abortion facilities in three Southeastern states, which have high rates of teen pregnancy, pronounced racial/ethnic and socioeconomic disparities, and multiple restrictions on abortion (Nash, 2019; National Academies of Sciences, 2017; U.S. Department of Health and Human Services & Office of Population Affairs, 2019). Two of the three states require in-person parental consent, and one requires parental notification. Two of the states require the parent to provide proof of their identity, with one requiring proof of relationship to the minor in the form of the patient’s certified birth certificate. In addition to these regulations, all three states require patients to receive state-mandated information about the risks and alternatives to abortion at least 24 h before attending their abortion visit; in one state, this information can be provided by phone. All three states prohibit abortion after 20- or 22-weeks’ gestation and prohibit coverage for abortion in state health insurance exchange plans and Medicaid, with limited exceptions (Guttmacher Institute, 2020c, 2020b). Our results fill a notable gap in the literature around parental involvement policies and how the layering of multiple restrictions adversely affects minors’ abilities to obtain high quality care.

## Methods

### Recruitment and Data Collection

Beginning in Spring 2018, we contacted clinic administrators and medical directors at facilities in three Southeastern states by email or telephone to inform them about the study. We provided a description of the study objectives and asked them to identify several members of their staff who might be willing to participate in a one-time interview about their experiences with their state’s parental involvement requirements and providing care to minor patients. To capture a range of perspectives about the implementation of parental involvement laws on service delivery throughout the process of providing care, we aimed to interview at least three staff members at each facility who were involved in scheduling, counseling, or administrative reporting, including clinic directors. Prior to conducting the interviews, clinicians familiar with the policy context based on their experience providing abortion care in the Southeast but who were not involved as study participants reviewed the guide to ensure questions and probes captured the types of encounters and processes involved serving minors. We revised the interview guide based on this feedback.

Between May 2018 and September 2019, we conducted semi-structured, in-depth interviews with facility staff in all three states. Because there are only 15 abortion-providing organizations in the region, we do not identify the specific states to protect participants’ confidentiality. After asking participants to describe their roles and responsibilities so we could tailor questions and probe for additional details later in the conversation, we asked them to walk us through the processes at their facility for documenting consent/notification, patients’ and parents’ reactions to the requirements, and challenges individuals, families, and staff had complying with these requirements. We also asked staff to discuss the frequency with which minors pursue a judicial bypass and their knowledge about and clients’ perspectives on the petition process and court hearings.

Members of the study team who had graduate-level training in public health, qualitative research method experience, and extensive knowledge of the abortion policy context in each state conducted the interviews in person; owing to scheduling difficulties, we conducted three interviews by phone. Participants provided their oral consent to participate and to have their interviews recorded. Interviews lasted between 28 and 78 min (median duration: 49 min), and participants also completed a short demographic survey. After audio recordings of the interviews were transcribed, we reviewed the transcripts for accuracy and removed identifying information. In our review, we also assessed the variability of responses and decided to stop data collection when we determined that additional participants would unlikely provide new information (i.e., data saturation) (Saunders et al., 2018). The institutional review boards at the University of Alabama at Birmingham and Emory University approved the study procedures.

### Analysis

We conducted a thematic analysis to examine staff experiences with and perspectives on state parental involvement policies and the ways in which these affect the process of providing care to minors considering abortion. The research team first reviewed transcripts from each state and developed a preliminary coding scheme consisting of deductive and inductive codes based on domains in the interview guide and interviewer notes. Five members of the team then independently coded five transcripts, meeting after each one to discuss coding consistency, refine coding definitions, and add inductive codes to capture new ideas that emerged in the data. Once the coding scheme was finalized, four members of the team divided the remaining transcripts using a double coding process: two people coded each transcript independently and then the pair met to compare the application of codes to transcript text and discuss discrepancies to reach consensus about the most appropriate code. All four coders also met regularly to discuss the coding process and ensure consistency across transcripts. We used Dedoose to code and organize the transcript data.

In our review of the coded data, we noted that many of the interview responses aligned with the IOM Quality of Care domains (Institute of Medicine & Committee on Quality of Health Care in America, 2001). In the next stage of analysis, we organized codes that corresponded to the domains of efficiency, patient centeredness, timeliness, and equity. We did not include the domains of safety and effectiveness because our study did not focus on the clinical aspects of care, for which these domains are most appropriate. We then summarized the main themes and assessed differences across states. Overall, the themes that emerged were similar across states, despite some differences in states’ parental involvement requirements and varied interpretations of certain regulations across facilities. We note differences as needed to add context to the presentation of results.

## Results

To capture geographic diversity, we reached out to 12 of the 15 organizations providing abortion care and for which we had contact information. Nine agreed to take part in the study; the facilities that declined to participate primarily noted their low volume of minor patients. Most of the participating facilities specialized in providing abortion care and reported that the majority of their patients were adult women of color who resided in the same state where the facility was located; minors and non-English-speaking patients typically accounted for < 10% of facilities’ clients.

A total of 34 staff members completed in-depth interviews across the three states. Nine staff members served as clinic directors or administrators, and other participants held positions as call center staff, counselors, or health educators, or were involved in other aspects of clinical care (Table 1). At some facilities, participants were involved in more than one aspect of scheduling, counseling, and reporting for minors seeking abortion. Most participants had worked in sexual/reproductive healthcare for more than 10 years, with the majority working continuously at the same facility at which they were interviewed.

### Efficiency

Because of patients’ diverse family histories and caregiving arrangements, all participants reported that their facility typically requested parents and guardians accompanying a minor at their initial visit for services to present documentation verifying each person’s identity, beyond what state statutes may have required. For example, staff at most facilities in all three states requested that parents bring the birth certificate as documentation to the visit, regardless of the state’s specific identification requirement, as well as marriage licenses or divorce decrees to prove the parent’s relationship to the minor in cases in which their last names differed. A receptionist in state 1 described this common practice, saying their facility needed “at least the minor’s birth certificate and at least the parent or guardian’s ID… If the parent’s ID says Jones and they were Wilson on the birth certificate, we ask for marriage, adoption, or divorce decrees as to why the name’s changed to be sure that they’re the same person.” Staff tried to identify such discrepancies when patients called to schedule an appointment; as a receptionist working at another facility relayed, “We try to prepare them really well over the phone… We ask them ahead of time like ‘Okay, is that the name that matches on the birth certificate?’ So they don’t waste their time.” Although a few facilities allowed the state-mandated consultation to proceed if a parent or guardian lacked clinic-required documentation so long as they returned for the abortion visit with the missing documents, families typically had to reschedule. Participants involved in such interactions commented that turning these families away was difficult because they may have driven several hours to obtain care.

The majority of facilities also asked for minors’ identification, such as a photo ID, as further evidence of the parent–minor relationship. An administrator in state 3 that required such identification explained to parents, “This birth certificate does not tell me that’s who’s standing right here… That doesn’t tell me that that’s her. I have to be able to see it and put a face with the name. The only thing this birth certificate tells me is that … you’re this person’s parent.” While staff preferred minors to present a state-issued ID, they recognized this was not always possible, particularly for those younger than 16 years of age. Thus, most clinics allowed for greater variability in the forms of identification that they accepted, as long as the materials associated the minor’s picture and name, such as school yearbooks. Participants noted that this process overall was “time consuming and not necessary,” and some commented that patient documents were unlikely to undergo the same level of scrutiny when minors sought other types of medical care, including those who decided to continue their pregnancies.

In an effort to minimize or eliminate the possibility of missing or unclear information about a patient’s age or the parent–minor relationship, participants in all three states described redundancies that had been established in their protocols for serving minors. This was largely related to their concerns and fears about receiving written citations from state inspectors or risk being shut down following state audits, with several participants noting that minors’ charts were the first ones selected for review during state health officials’ regular compliance inspections. Beginning with the initial call for an appointment, staff involved with scheduling said they spend “a lot of time on the phone” explaining the parental involvement requirements and “reiterate two or three times [the documents they need to bring] when we’re on the phone”, and asking parents and minors to repeat the information back to them. Most facilities also frequently had staff in various roles, such as registration and reporting, confirm that patients’ ages matched dates of birth, that documentation of identification was complete, and that all forms had the patients’ and parents’ signatures where needed. For example, after being cited for a discrepancy between the age and birthdate for a minor patient who had parental consent, an administrator in state 2 noted that consent and documentation are checked at registration for the consultation visit and then re-checked before the abortion visit can take place. She explained, “I have changed my paperwork so that I have to initial off that I have literally looked at the date of birth for this minor, matched it up with what she told us it was, then matched it up with an ID, if I have the ID. Then, of course, I always have to look at the parent… If my initials aren’t on it, that chart doesn’t go through anywhere.” As a result of these checks and balances, staff reported it could take twice as long to complete paperwork for minor patients, given other minor-specific reporting for statutory rape and abuse if the patient is younger than 16, as well as state-mandated informed consent policies and facility-specific consents for medication or procedural abortion, both of which are required for minor and adult patients. Expressing the frustration of many participants about the extensive reporting and documentation required for minors, an administrator in state 1 stated, “Even if the state isn’t going to trust young women who are having sex and get pregnant, trust the clinics that are here because this is what we do every day.”

### Patient-Centered Care

Clinic staff universally recognized the need to tailor services in order to address minor patients’ different developmental needs and life experiences. Compliance with state restrictions added complexity to staff efforts to accommodate these differences and still try to provide patient-centered care throughout each step of the process. All staff involved in patient scheduling who explained parental involvement requirements over the phone commented that they would spend additional time talking with some minors who seemed hesitant to involve a parent to help them identify which mechanism (consent/notification or bypass) might work best for their circumstances. Similar to others who acknowledged minors’ concerns about disclosing their pregnancy to a parent, a call center manager in state 1 described how she walked patients through their options, “If it’s a situation where she feels threatened … to tell her mom or dad that she’s pregnant, you know, definitely I let them know about the judicial bypass process. If it’s a situation where she’s just afraid…I kind of talk to her and let her know, ‘It’s okay, maybe you can call back with mom on the phone, if you’re comfortable with that.’ So it just depends on the situation.” When minors informed staff that they were going to pursue a judicial bypass, participants reported spending additional time on the phone and at in-person clinic visits preparing these patients to answer questions that a judge was likely to ask at a hearing: reasons for not wanting to involve a parent, understanding of the procedure, and even plans for post-abortion contraception.

In all three states, participants pointed out that even when minors arrived at visits with a parent, this did not mean parental involvement was welcome or that the parent was supportive of an abortion decision. A counselor in state 3 was one of the many participants who called attention to this issue, saying, “Some don’t seem to be happy that their parents came. They have made up their minds that they want to have an abortion… but they don’t want their parent involved in it really.” Nearly all participants recounted a time when they felt a duty to intervene with a parent to ensure the minor’s preferences remained centered. In some cases, this involved a parent who attended the visit but did not provide emotional support, and instead was “shaming and guilting” and being “emotionally harmful.” A counselor in state 2 recalled stepping in during one such exchange and pulling the parent aside to say, “Now is not the time for you to scold her. Now is the time for you to be there for her… Now she needs some help, so that should be your main focus… helping her.” They also reported times when minors themselves were hesitant or did not want to get an abortion, and staff at all facilities made clear that the visit would not proceed if there was any doubt about a minor’s desire for abortion. An administrator at another facility in state 2 described a typical practice and conversation for handling these kinds of situations if they occurred prior to meeting one-on-one with the provider, explaining, “We separate the minor from the parent then talk to the minor… If she says, ‘No [I don’t want an abortion].’ then I’m obligated to advocate for the patient.”

In order to address minors’ varied personal circumstances, clinic staff said that they were willing to “bend over backwards” to ensure these patients got the services they needed. This included offering minors individualized (versus group) counseling sessions because it allowed them to be more forthcoming about any concerns regarding their relationships or pregnancy desires. However, a few participants expressed that this placed pressure on facility staff as they worked to accommodate the many other patients needing care. An administrator in state 1 acknowledged this tension saying, “While we want to spend as much time [as we can]… Ultimately, that’s a really hard thing to do because [we] have 16 clients that are waiting to be seen in the morning.”

### Timeliness

Participants in all three states reported that parental involvement laws and other state abortion restrictions, combined with clinic protocols for ensuring compliance, often delayed minors throughout the process of obtaining an abortion, and these delays could add days or weeks to patients’ timeline of receiving care. Many staff stated that minors initiating contact with the facility “usually know that there is some kind of process” involved for getting an abortion but can get overwhelmed after learning what they need to do. A counselor who also helped with scheduling in state 3 acknowledged that, while most minors expect to involve an adult, others can be put off after learning that a parent needs to come to their visit, “They’re shocked. They go away and… we don’t know if they ever go through the actual process.” A receptionist working at a facility in state 1 shared these concerns, explaining this was often the case for minors who had not yet disclosed their pregnancy, “When… they call us initially, they might be a lot earlier on, but then, when we tell them they have to have their parent involved—or a letter of notification, [that] scared them too, because they don’t want a letter being sent to their parent’s house—or the judicial bypass. You know, it’s very invasive and intimidating for a minor… I think it definitely proves to deter minors from getting the abortion care they might need a lot earlier.”

Many participants also commented that care could be delayed when parents called to schedule their minor’s appointment since facilities typically required both the parent and minor-patient to be on the phone to confirm the minor wanted an abortion and to relay the state requirements. Although requiring a return call could postpone scheduling and frustrate parents who were accustomed to independently arranging other medical visits, staff explained that having both parties on the phone helped avoid other problems that delayed care. In the state allowing the state-mandated information about abortion to be provided by phone, a receptionist recalled delays that occurred when staff would schedule an appointment but the caller’s parent or teen did not call back as requested to hear the information: “They present themselves on that day, but the parent never called back or the daughter never called back. So, in a sense, they weren’t read the ‘Women’s Right To Know’… So, of course, the process stops again there because they’re not able to go ahead and complete [the visit].” Additionally, if parents needed to request an out-of-state birth certificate or if they did not arrive with the necessary documentation proving their relationship to the minor, it could be another week and as long as a month before they had the required paperwork and could return to the clinic.

Other legal avenues for minors to obtain an abortion also contributed to delays in care. For example, one state permitted parental notification, but required a 48-h waiting period after mailing the notification letter before the abortion visit could take place. This timeline could be extended if the post office determined the address was invalid and was unable to issue the confirmation receipt that staff attach to the patient’s chart. A minor using parental notification may wait more than 48 h to attend their abortion visit because of the limited availability of appointments. As a receptionist noted, this can push minors up against the clinic’s or state’s gestational age limit, “If a patient calls on a Friday afternoon, like we’re not going to be able to get this [to the post office] until next week. So, you know, that puts you off three or four days. And if you’re in your second trimester, you know, it’s time consuming.”

Additionally, most staff with experiences with the judicial bypass agreed that the process frequently prolonged the time it took for a minor to return for the abortion visit. The process in larger counties, where clinics were typically located, was seen as the most expeditious, but participants across all three states noted it could still take up to a week for a minor to comply with state-mandated informed consent, file the bypass petition, get a hearing, and then return to the clinic. In smaller counties, the process could take 2 weeks or longer owing to court staff’s lack of familiarity with the process and more conservative attitudes about abortion. A few participants recounted minors telling them that court staff refused to give them the paperwork to file a petition and judges who “just didn’t believe in doing [abortions] at that stage of pregnancy.” A receptionist working in state 2 recalled a case from the prior year in which a minor tried to get a bypass in her county of residence, but the courthouse staff “were putting her off.” She finally obtained a bypass in the county where the clinic was located and arrived for her visit in the late afternoon. The respondent reported, “We were through with appointments, but because she had had such a hard time [and] the nurse was still here and the girls were in the back, we decided we’d let her … come in. And she ended up being too far. She was further along than our doctors go.”

### Equity

Across all three states, staff discussed how the burdens of compliance with parental involvement policies fell more heavily on some minors because of their social or economic circumstances, which further compromised access to timely care. For example, the cost of obtaining an original birth certificate or photo identification could be difficult for lower income families that were already struggling to pay out of pocket for an unanticipated medical expense because abortion was not covered by Medicaid and some private insurance plans. Several participants noted that paying $15 to $20 to obtain these documents could be the cost of gas some families needed to get to the facility. A receptionist in state 3 pointed out the financial difficulties associated with getting documentation and how this could push minors over the facility’s gestational age limit, “It’s hard for them to come up with the money to get the IDs ‘cause they have to pay for the surgeries. They’re like, ‘I don’t have the extra money to go get an ID made’. Sometimes that’s a set-back for them. Or they don’t have a birth certificate – they have to go get one made… It pushes them to where they might go over 16 weeks trying to get that paperwork.” A health educator who assisted with scheduling at a facility in state 1 added that requesting and obtaining birth certificates from state vital statistics offices also could be logistically challenging for families who did not have regular computer access, whose minor was born out of state or outside the USA, or whose schedules did not align with regular business hours. A nurse working at another facility in state 1 echoed this sentiment saying, “Then, having to pay money to go get another copy [of the birth certificate] or drive down to the vital records office to get another copy. They have to take time off from work. What if they work two jobs? What if they’re working nights?”.

Minors with more complex family circumstances had a particularly convoluted path to obtaining an abortion. Participants at all facilities cited instances in which it was difficult for families to provide the necessary documentation because a caregiver had informal custody following a parent’s death, incarceration, or struggle with substance use. Noting the many cases of grandmothers coming to their facility with grandchildren they had been raising since birth, an administrator in state 1 said, “The impact comes when the person doesn’t have the documentation that they’re required to get and trying to direct them through the maze to find certain things in order to prove other things.” A few participants commented that families might present documentation from a state agency showing a fostering arrangement or paperwork indicating the adult could serve as a medical proxy for the teen, but staff did not believe this would be sufficient for their reporting purposes. Although the adults served as a de facto guardian, without official paperwork, these families would be referred for a judicial bypass, which could further extend the minor’s timeline to get an abortion. These complex arrangements also could confuse courthouse staff. An administrator from state 2 recounted a case in which a mother had relinquished custody of her daughter (the minor seeking care) to her parents and changed the minor’s birth certificate, but later began parenting when her father died and her mother entered hospice care. Unable to accept the birth certificate, staff at the clinic referred the family to the courthouse to petition for a bypass and recalled, “I was so frustrated sending them to intake, and then them [courthouse staff] sending them away telling them they don’t need a judicial bypass because she is the mother. ‘Well, yeah. She is the mother, but I don’t have proof of that. They have to go before a judge’ and—I sent them back over… They were from 100 miles away—judicial bypass was bad enough, much less for me to keep sending them 15 min over [to the courthouse].”

## Discussion

Abortion facility staff in this study discussed multiple ways that parental involvement requirements adversely affected health service delivery and quality of care for minors seeking abortion in their states. Our findings expand on prior literature about the impact of parental involvement requirements (Dennis et al., 2009; Janiak et al., 2019; Kavanagh et al., 2012; Ralph et al., 2018) by focusing on the processes through which these restrictions affect care for the many minors who involve a parent and obtain in-state services. These results are also consistent with conclusions reached by the National Academies of Sciences (National Academies of Sciences, Engineering, and Medicine, 2018) that abortion restrictions decrease efficiency, disregard patient preferences, delay time-sensitive care, and are more burdensome for some patients who may already experience socioeconomic disadvantages.

In particular, participants highlighted that parental consent and notification are far more cumbersome than simply documenting that a parent is aware of and agrees to the minor’s decision to have an abortion. Facilities have developed intensive and redundant documentation processes to ensure they have adequate evidence demonstrating an adult’s legal relationship to a minor. This may be related to vague statutory language about necessary documentation and the fact that identification requirements offer little flexibility for diverse family structures and living arrangements. Additionally, facility staff may be concerned about getting cited or shut down for even minor deviations from state requirements, based on past experience or anticipated hostility during regular state audits of minors’ charts. Such intensive and unwarranted monitoring of abortion care for minors provides another example of how state agencies may be enforcing regulations that are not consistent with public health principles (Roberts et al., 2017) and lead to inefficient care. Additionally, the US South has one of the highest shares of grandparent caregivers, many of whom are Black and living on low incomes (Clottey et al., 2015; Whitley et al., 2007). Youth and families in these circumstances are already facing social disadvantage due to systemic racism and other discriminatory policies that contribute to disproportionate rates of economic instability, incarceration, and substance abuse, and the documentation requirements further penalize them by placing considerable burdens to prove legal guardianship. As participants also noted, families’ challenges meeting documentation requirements can delay care and unnecessarily divert families to burdensome judicial bypass proceedings, even when a supportive caregiver is involved.

Our findings also indicate that parental involvement policies can pose challenges for staff committed to offering patient-centered care and do not inherently foster constructive parental engagement. Participants acknowledged that the minors they serve are making well-thought-out reproductive decisions, as reported elsewhere (K. Coleman-Minahan et al., 2020; Erlich, 2003) but then are forced to navigate an emotionally and logistically burdensome pathway to abortion. Given the more onerous and potentially traumatic process of pursuing a judicial bypass (K. T. Coleman-Minahan et al., 2019; Kavanagh et al., 2012), staff often focused minor-initiated calls around parental consent (or notification) to assess whether this mechanism would be feasible. When minors have clear reasons for not wanting to tell a parent, they often involve a trusted adult in their abortion decision (K. Coleman-Minahan et al., 2020; Erlich, 2003; Hasselbacher et al., 2014; Henshaw & Kost, 1992). However, those adults, without official decision-making rights, are unable to consent or to receive notice for a minor’s abortion decision. Moreover, in instances when an involved parent was unsupportive of the minor’s decision, participants later found themselves serving as a mediator to ensure the young person’s desires for reproductive autonomy remained central, adding additional demands for staff who typically held multiple roles.

This study also lends further evidence to prior research that parental involvement laws, like other abortion restrictions (e.g., mandatory waiting periods), not only fail to serve their stated purpose but also can delay patients obtaining care (Ellertson, 1997; Joyce et al., 2006; Karasek et al., 2016; Ralph et al., 2018, 2021; Roberts et al., 2019). Participants we interviewed pointed to the ways in which these laws operate in concert with other restrictions to prolong minors’ timeline for having an abortion, which may have already been delayed by late recognition of pregnancy, reservations about pregnancy disclosure, difficulties getting to a clinic, and paying for care. These delays are even more concerning given the state-imposed and clinic-level gestational age limits that narrow the timeframe in which minors can get an abortion and may force some to continue unwanted pregnancies. The constrained options for timely, patient-centered care created by the elaborate architecture of restrictions lies in sharp contrast to the Society of Adolescent Health and Medicine and American Academy of Pediatrics’ recommendations that comprehensive and confidential reproductive healthcare services, including abortion, should be accessible for minors (American Academy of Pediatrics, 2017; The Society for Adolescent Health and Medicine & American Academy of Pediatrics, 2014).

To support higher quality care, policies surrounding abortion for minors need to be fundamentally re-envisioned and not based on assumptions of nuclear families and universal benefits of parental involvement. This would involve removing the burdens of unnecessary paperwork and trusting that abortion providers, like other medical professionals, have their patients’ best interests in mind. Moreover, policies should acknowledge that people seeking abortion care, including minors, are capable of making autonomous decisions around their reproductive health. For minors who are beginning to take responsibility for their own health care, this would mean allowing them to decide who to involve in their abortion decision, instead of imposing rigid requirements that could place them in daunting and demeaning situations. These changes, together with those repealing mandatory waiting periods for all patients, would allow abortion providers more flexibility to tailor services to minors’ unique needs and familial circumstances and would better fulfill professional organizations’ recommendations for quality patient-centered care.

### Limitations

A limitation of our study is that participating organizations were located in a single region of the USA and not all of those invited to participate took part in the study. While our results may not be generalizable, they likely reflect the service environment in this area because staff from the majority of facilities participated and our results were largely consistent across facilities that were diverse with respect to client volume and communities served. Additional research is needed to confirm whether these effects on quality of care occur in other settings that also have parental involvement laws but fewer overall restrictions on abortion. We also do not have client reports on time to care or data on gestational age that would support participants’ claims that parental involvement policies delay minors seeking abortion care, but their perspectives are consistent with some prior studies that have assessed these outcomes (Ellertson, 1997; Joyce et al., 2006; Ralph et al., 2021). Moreover, these findings offer new insight into some of the factors that may contribute to these delays. Finally, we do not know if the ways in which staff portrayed the challenges of compliance correspond with the experience of parents, guardians, and minors seeking care in these settings. Further research assessing additional perspectives on navigating these requirements is needed to provide a comprehensive view of how these policies impact care.

## Conclusions

Our data reveal that parental involvement laws, which are among the many layers of abortion restrictions in these states, impede high-quality care for minors by creating inefficiencies in clinic operations, undermining patient preferences, needlessly delaying care, and exacerbating structural inequities. These effects may not only stem from the existing parental involvement statues but the broader overregulation of abortion care that likely has contributed to facilities developing protocols requiring additional documentation to ensure compliance during state audits. The laws’ requirements also undermine the professional medical judgment of abortion providers and impose unnecessary burdens on their delivery of care. Removing the multiple restrictions on abortion would better serve patients, regardless of age, and providers’ abilities to offer this essential health service. Public health professionals and policy makers should advocate for evidence-based laws that protect reproductive autonomy, informed medical decision-making, and promote equitable, patient-centered quality of reproductive healthcare for all patients.

## Figures and Tables

**Table 1:** Selected characteristics of abortion facility personnel in three Southeastern states, 2018–2019

Characteristic	Number (*n* = 34)
Age, years	
24–34	4
35–44	10
45–54	10
≥ 55	10
Role at clinic	
Director/Administrator	9
Support Staff	18
Clinical/Patient Care	7
Years working in sexual/reproductive health	
< 3	3
3–9	10
≥ 10	21
Years in current position	
< 3	11
3–9	12
≥ 10	11
Years at current organization	
< 3	11
3–9	10
≥ 10	13
